# GPR161 mechanosensitivity at the primary cilium drives neuronal saltatory migration

**DOI:** 10.1126/sciadv.adx3846

**Published:** 2025-07-30

**Authors:** Théo Paillard, Ada Allam, Mohamed Doulazmi, Mathieu Hautefeuille, Coralie Fouquet, Liza Sarde, Julie Stoufflet, Salima Messaoudi, Nathalie Spassky, Stéphane Nédélec, Isabelle Dusart, Alain Trembleau, Isabelle Caillé

**Affiliations:** ^1^ Sorbonne Université, CNRS, INSERM, NeuroSU, F-75005 Paris, France.; ^2^ Sorbonne Université, CNRS, INSERM, Institut de Biologie Paris Seine, IBPS, F-75005 Paris, France.; ^3^ Sorbonne Université, CNRS, INSERM, Dev2A, F-75005 Paris, France.; ^4^ Institut Curie, CNRS, Sorbonne Université, Physique des Cellules et Cancer, Paris, France.; ^5^ PSL Research University, Ecole Normale Supérieure, CNRS, INSERM, Institut de Biologie de l’Ecole Normale Supérieure, Paris, France.; ^6^ Université Paris Cité, CNRS, Institut Jacques Monod, Paris, France.; ^7^ Institut Jacques Monod, INSERM U1340, Paris, France.

## Abstract

The saltatory migration of neurons is essential for brain formation. Whether mechanical stimuli regulate this process is unknown. Here, we show that the primary cilium acts as a mechanical sensor through GPR161. Using an ex vivo neuronal migration model and microfluidic assays, we demonstrate that fluid shear stress induces migration via the mechanoreceptor GPR161 at the primary cilium, with its mechanosensitive helix 8 being essential. We demonstrate that GPR161 activates a recently discovered cAMP/PKA signaling pathway leading to the phosphorylation of NDE1, a dynein complex regulator, and microtubule organization to regulate migration. These findings unveil a critical role of mechanosensation in neuronal migration, regulating the rhythmicity of migration, in concert with the externalization/internalization dynamics of the primary cilium.

## Teaser

Primary cilium senses mechanical cues to regulate young neuron migration via GPR161 signaling.

## INTRODUCTION

**Fig. 1.**
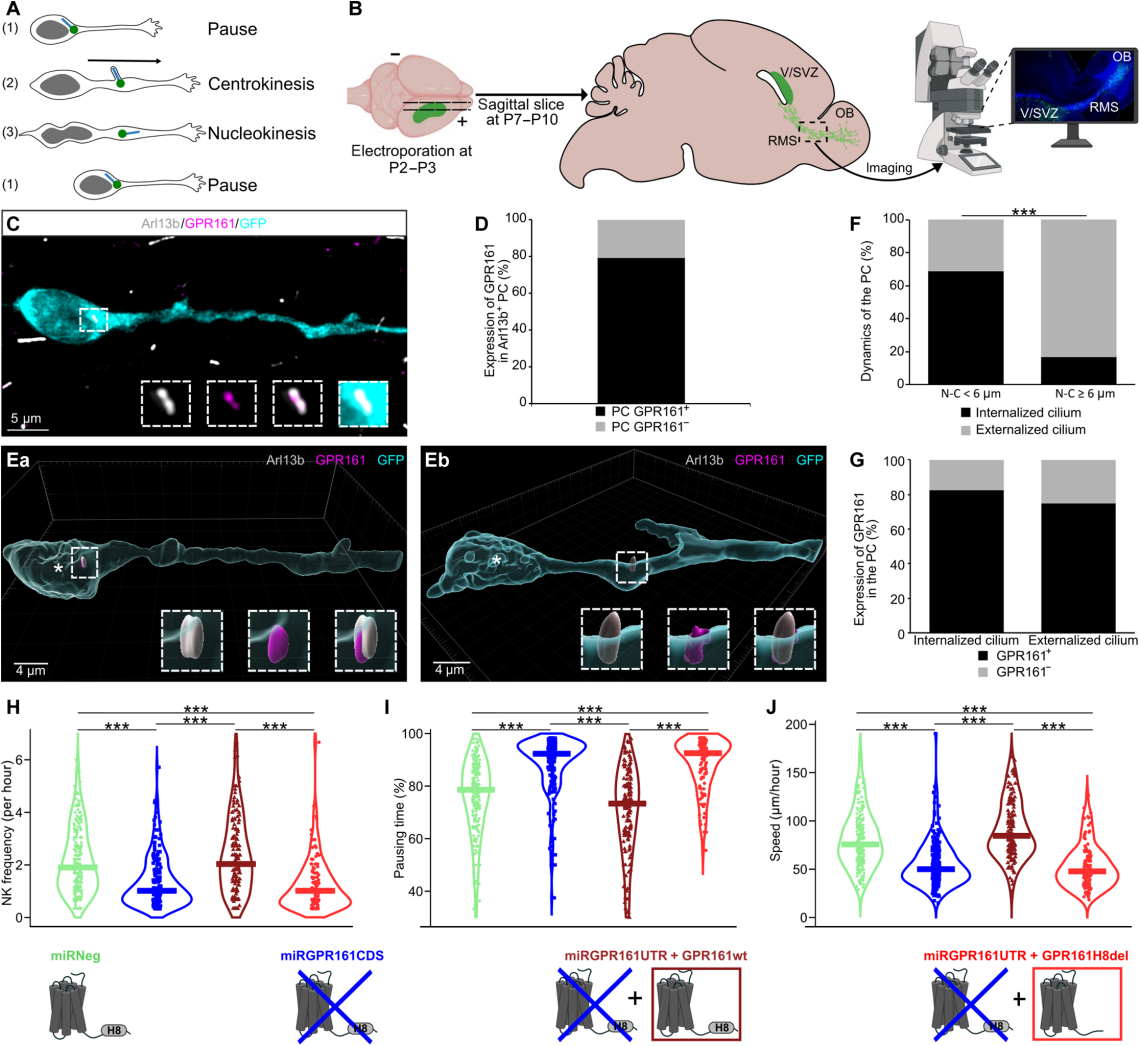
GPR161 regulates migration via its helix-8. (**A**) Cyclic saltatory migration steps of a neuroblast. (1) Pause: Centrosome (green dot) next to the nucleus (gray), PC (blue line) internalized. (2) CK: Movement of the centrosome within a swelling in the leading process, PC externalized. (3) NK: Nucleus movement, PC internalized. (**B**) Experimental procedure: Pups were electroporated at postnatal day 2 (P2) to P3, their brain was taken at P7-P10, and imaging of slices was performed. (**C**) Immunohistochemistry of a GFP-positive RMS neuroblast (cyan) showing GPR161 subcellular immunolabeling (magenta) in the Arl13b-positive PC (gray). (**D**) Percentage of GFP^+^ RMS neuroblasts presenting a GPR161^+^/Arl13b^+^ or GPR161^−^/Arl13b^+^ immunoreactive PC in miRNeg electroporated neuroblasts (*N* = 3, *n* = 51). (**Ea** and **Eb**) 3D reconstructions of migrating neuroblasts using Imaris software [immunostaining performed as in (C)]. White asterisk indicates the nucleus center. The distance from the nucleus-to-cilium base is less than 6 μm in (Ea) and greater in (Eb). The PC (Arl13b positive, gray) is GPR161 positive (magenta) and internalized into the cytoplasm in (Ea) and externalized at the cell surface in (Eb). (**F**) Percentage of internalized and externalized PC in neuroblasts in a pausing time morphology and a migrating morphology based on the nucleus-to-cilium (N-C) distance. During pausing time (N-C < 6 μm), the PC is predominantly internalized. During migrating phase (N-C ≥ 6 μm), the PC is predominantly externalized (*N* = 3, *n* = 53). Pearson’s χ^2^ test (1, *N* = 53) = 14.50, *P* < 0.001. (**G**) Percentage of Arl13b^+^/GPR161^+^ or Arl13b^+^/GPR161^−^ immunoreactive PCs in their internalized and externalized states (*N* = 3, *n* = 51). Pearson’s χ^2^ test (1, *N* = 51) = 0.432, *P* = 0.51. (**H** to **J**) Rhythm of migration (see also fig. S5): (H) NK frequency per hour, (I) percentage of neurons in pause, (J) speed of migration (micrometers per hour) in neuroblasts electroporated with miRNeg (green), miRGPR161CDS (dark blue), miRGPR161UTR + GPR161wt (dark red), or miRGPR161UTR + GPR161H8del (bright red). *P < 0.05, **P < 0.01, ***P < 0.001.

During development, vertebrate neurons migrate from their site of birth to their final destination. Neuronal migration defects lead to severe brain malformations associated with neurological or psychiatric disorders (*1*–*3*). Migrating neurons exhibit a saltatory pattern of movements, characterized by alternating phases of somal translocation and pauses (*4*). They display a pattern of successive cellular events including the extension of a leading process in the direction of the migration, a movement of the centrosome within this process [centrokinesis (CK)], followed by nuclear translocation [nucleokinesis (NK)] and a pause, before the whole process resumes (Fig. 1A) (*4*, *5*). The primary cilium (PC), a microtubule-based organelle emanating from the centrosome, was recently shown to regulate the directionality and/or dynamics of migrating neurons (*6*–*11*). The PC itself displays a cyclic behavior, through externalization during CK, and internalization during pauses (Fig. 1A) (*6*, *10*). The PC also acts as a dynamic and cyclic source of cyclic adenosine 3′,5′-monophosphate (cAMP) that accumulates at the centrosome during the CK and NK, where it activates protein kinase A (PKA) (*11*). Interfering with either cAMP production or PKA centrosomal localization decreases the speed of migration with longer pauses and less frequent NK. Therefore, the PC produces cAMP when it is externalized upon CK, and the resulting PKA activation at the centrosome influences downstream targets that regulate the cytoskeleton (*12*). In recent years, the PC’s ability to deflect in response to mechanical stimuli has raised speculation about its mechanosensory functions. Recent studies provided compelling evidence that the PC plays a mechanical role in the left-right organizer to instruct left-right asymmetry (*13*, *14*). Yet, whether PC-mediated mechanosensitivity plays a role during nervous system development is unknown.

This possibility was supported by the recently described presence of the mechanosensitive orphan Gαs-coupled receptor GPR161 (*15*) in the PC of neurons (*16*). We therefore investigated whether neuronal migration may be mechanically regulated through GPR161-dependent signaling pathways.

## RESULTS

### GPR161 regulates neuronal migration via its mechanosensitive helix 8

The postnatal migration of neuroblasts from the ventricular/subventricular zone (V/SVZ) is a well-established model for studying neuronal migration. In rodents, V/SVZ-derived neuroblasts display a cyclic saltatory migration (Fig. 1A) as they traverse the rostral migratory stream (RMS) to reach the olfactory bulb (OB; Fig. 1B) (*17*). A few days after intraventricular electroporation of a green fluorescent protein (GFP)–expressing plasmid in neonate mice, a cohort of migrating neuroblasts can be visualized in acute slices of the RMS through GFP fluorescence (Fig. 1B).

To investigate whether GPR161, a mechanoreceptor, has a role in neuronal migration, we first confirmed that GPR161 is expressed by V/SVZ-derived neuroblasts and localized in the PCs in vivo (Fig. 1, C and D). In GFP^+^ neuroblasts in the RMS, 79.3% of PC stained by Arl13b, a PC marker (*18*), were also GPR161 immunoreactive (Fig. 1D). In addition, three-dimensional (3D) reconstructions revealed a dynamic PC localization in migrating neuroblasts: it is either internalized (Fig. 1Ea and movie S1) or externalized (Fig. 1Eb and movie S2). As previously described (*10*), it is internalized during the pausing phases (when the PC is near the nucleus) (69.0%) and externalized during CK [defined here as when the nucleus-to-PC distance is superior or equal to 6 μm; (*11*)] (83.3%) (Fig. 1F). GPR161 is consistently present in the PC, regardless of its internalization/externalization state (respectively, 82.6 and 75.0%) (Fig. 1G).

We next used an interfering RNA targeting GPR161 mRNA coding sequence (miRGPR161CDS; figs. S1A and S2) to down-regulate GPR161 expression in V/SVZ-derived neuroblasts. As controls, we used an interfering RNA predicted not to target any known vertebrate mRNA (miRNeg). The efficiency of the knockdown was assessed by GPR161 immunostaining on RMS migrating neuroblasts on brain slices. While 79.3% of neuroblasts electroporated with miRNeg plasmid were displaying an Arl13b and GPR161 immunoreactive PC, this was significantly reduced to 38.3% in miRGPR161CDS electroporated neurons (fig. S2). Five to 7 days after transfection, we imaged the behavior of neuroblasts in acute brain slices of the RMS (miRNeg: movie S3; miRGPR161CDS: movie S4). The parameters of the rhythm of migration (NK frequency, pausing time, and migration speed) were analyzed only for the neuroblasts performing at least one NK [i.e., a nuclear movement superior or equal to 6 μm; (*11*)]. We detected a significant reduction in NK frequency [expressed as median (interquartile range): miRNeg: 1.9 (1.8) NK/hour versus miRGPR161CDS: 1.0 (1.1) NK/hour] (Fig. 1H), a significant increase in pausing time [miRNeg: 78.6% (19.3); miRGPR161CDS: 92.3% (10.2)] (Fig. 1I) and a significant decrease in migration speed compared to miRNeg [miRNeg: 75.7 (36.6) μm/hour; miRGPR161CDS: 50.0 (26.9) μm/hour] (Fig. 1J). These data demonstrate that GPR161 is essential for proper neuronal migration.

GPR161 has been described as mechanosensitive (*15*) and has a helix 8 (*19*, *20*), a C-terminal intracellular domain necessary and sufficient for GPCR mechanosensitivity (*21*). To test whether the GPR161’s role in migration depends on its mechanosensitivity, we investigated whether the helix 8 of GPR161 is required for its role in neuronal migration. We created plasmids expressing either the wild-type GPR161 coding sequence (referred to as GPR161wt) or GPR161 lacking its helix 8 (GPR161H8del) plasmids (fig. S1, B and C). To down-regulate the endogenous GPR161, but not the exogenous constructs, pups were coelectroporated with GPR161wt or GPR161H8del along with a miRGPR161 targeting specifically the GPR161 3′ untranslated region (miRGPR161UTR) absent from our plasmids sequence (fig. S1, B and C). The knockdown efficacy of the miRGPR161UTR was similar to that of the previously used miRGPR161CDS (around 50% efficiency) (fig. S2). Notably, the overexpression of the wild-type form of GPR161 in miRGPR161UTR knockdown neuroblasts (miRGPR161UTR + GPR161wt condition; movie S5) rescued all the migration parameters [NK frequency: 2.0 (2.0) NK/hour; pausing time: 73.3% (21.7); speed: 84.6 (39.0) μm/hour] (Fig. 1, H to J). In contrast, the overexpression of the GPR161 lacking the mechanosensitive helix 8 (miRGPR161UTR + GPR161H8del condition; movie S6) failed to rescue NK frequency, pausing time, and speed phenotype induced by the endogenous GPR161CDS knockdown [NK frequency: 1.0 (1.0) NK/hour; pausing time: 92.5% (12.0); speed: 47.8 (27.0) μm/hour] (Fig. 1, H to J). These findings demonstrate that GPR161 is a key regulator of neuronal migration through its helix 8, suggesting that mechanosensing is implicated in neuronal migration rhythmicity.

### Fluid shear stress induces neuronal migration through GPR161

**Fig. 2.**
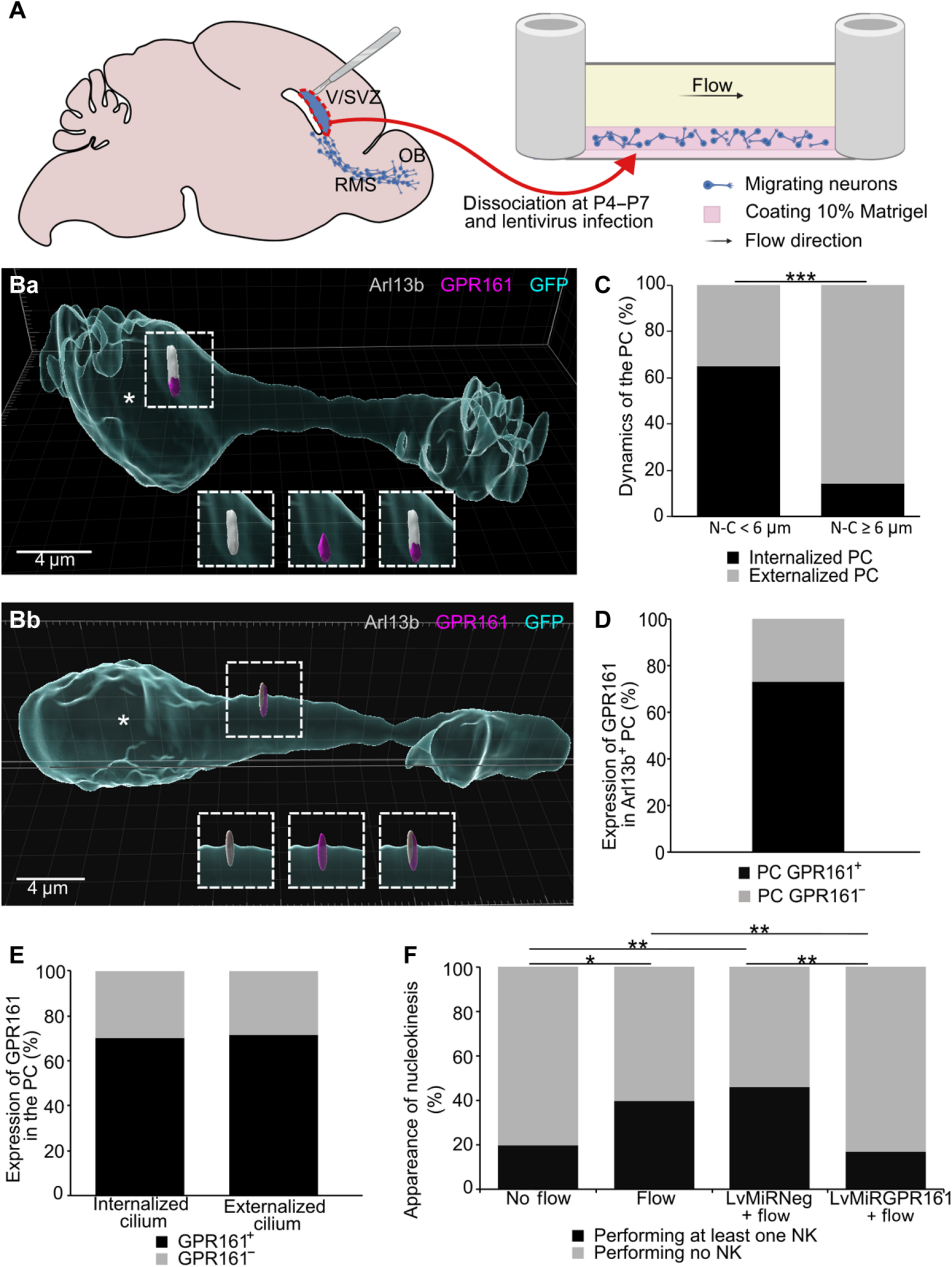
Fluid shear-stress induces migration through GPR161. (**A**) Experimental procedure: The V/SVZ, source of migrating neuroblasts in the RMS toward the OB, was dissected at P4 to P7. Cells were dissociated and infected or not with lentivirus and then plated in a microfluidic setup coated with 10% Matrigel. A flow was or not applied. (**Ba** and **Bb**) 3D reconstructions of migrating neuroblasts using Imaris software. The white asterisk indicates the center of nucleus. The distance from the nucleus-to-cilium base is less than 6 μm in (Ba) and greater than 6 μm in (Bb). The PC (Arl13b positive, gray) is GPR161 positive (magenta) and internalized into the cytoplasm in (Ba) and externalized at the cell surface in (Bb). (**C**) Percentage of internalized and externalized PC in neuroblasts in a pausing time morphology and a migrating morphology based on the distance nucleus-to-cilium (N-C). During pausing time (N-C < 6 μm), the PC is predominantly internalized. During migrating phase (N-C ≥ 6 μm), the PC is predominantly externalized at the cell surface (*N* = 5, *n* = 44). Pearson’s χ^2^ test (1, *N* = 44) = 11.78, *P* < 0.001. (**D**) Percentage of 2D-cultured neuroblasts displaying an Arl13b and GPR161 immunoreactive (GPR161^+^) or not (GPR161^−^) PC (*N* = 3, *n* = 26). (**E**) Percentage of Arl13b and GPR161 immunoreactive (GPR161^+^) or not (GPR161^−^) PC in its internalized and externalized states (*N* = 3, *n* = 24). Pearson’s χ^2^ test (1, *N* = 24) = 0.006, *P* = 0.94. (**F**) Percentage of neuroblasts performing at least one NK during the entire movie experiment (2.5 hours) in no flow (*N* = 3, *n* = 71) condition, flow condition (*N* = 3, *n* = 75), LvmiRNeg + flow (*N* = 3, *n* = 77), or LvmiRGPR161 + flow (*N* = 3, *n* = 65) conditions, with a flow corresponding to a shear stress of 0.13 Pa. Pearson’s χ^2^ test (1, *N* = 142) = 14.30, *P* = 0.01. *P < 0.05, **P < 0.01, ***P < 0.001.

We then sought to directly test whether mechanical forces influence neuronal migration. As fluid flow–associated shear stress mechanically influences PC dynamics (*22*), we developed a microfluidic device to expose dissociated neuroblasts to controlled fluid shear stress. Neuroblasts isolated from the V/SVZ of neonatal mice [postnatal day 4 (P4) to P7] were plated on a 2D Matrigel-coated substrate within a channel connected to pumps that generate a fluid flow (Fig. 2A).

In this setup, as in brain slices (Fig. 1), neuroblasts had a typical leading process with a PC revealed by the presence of Arl13b immunostaining (Fig. 2, Ba to Bb, and fig. S3A). 3D reconstructions indicate that neuroblast PCs retain their typical externalization/internalization dynamics (Fig. 2, Ba and Bb, and movies S7 and S8). As in vivo in the postnatal brain (Fig. 1) (*10*), PCs of neuroblasts in our 2D cultures are internalized during pausing phases in 65.2% of neuroblasts and externalized during CK in 85.7% of neuroblasts (Fig. 2C). GPR161 immunoreactivity was also detected in 73.1% of Arl13b-positive cilium (Fig. 2D) and remained consistently present in the PC, whether it was internalized or not (70.0% GPR161^+^ in internalized PC and 71.4% GPR161^+^ in externalized PC; Fig. 2, Ba, Bb, and E). Therefore, neuroblasts cultured in 2D in microfluidic chambers retain the subcellular localization of GPR161 and the dynamics of their PC as ex vivo. We, therefore, used live imaging in this microfluidic chamber to address the effects of fluid flow–induced mechanical stimulation on neuroblast migration.

Under no flow condition, only a small subset of neuroblasts displayed migratory behavior (movie S9), with only 19.7% of the neuroblasts performing one or more NK events over the 2.5-hour time period of imaging (Fig. 2F). In contrast, a fluid flow that generated a 0.13-Pa shear stress doubled the number of migrating neuroblasts, with 40.0% of the cells displaying one or more NK events (Fig. 2F and movie S10). Analysis of the directionality of NK revealed that this parameter is independent of both the expression of GPR161 and the direction of the flow. In the flow conditions, neuroblasts did not migrate more in the flow direction, demonstrating that the migration induced by the fluid flow is not due to a displacement of the neuroblasts by the flow (fig. S3B). Hence, fluid shear stress is sufficient to promote neuronal migration. To determine whether GPR161 is responsible for the fluid flow–dependent activation of migration, we designed a lentivirus coexpressing GFP with either the interfering RNA targeting GPR161 mRNA coding sequence (LvmiRGPR161) or the miRNeg (LvmiRNeg). Upon LvmiRNeg infection under flow conditions, neuroblasts displayed the same percentage of cells performing NK as in the absence of infection (46.2%; Fig. 2F and movie S11). However, GPR161 knockdown blocked the flow-induced migration (16.9%; Fig. 2F and movie S12). Together, these results demonstrate that the migration of V/SVZ neuroblasts is regulated by mechanical stimuli and that mechanosensitivity is mediated by GPR161 located at the PC.

### GPR161 regulates the organization of the nuclear cage of microtubules in migrating neuroblasts

We next sought to identify the mechanosensing pathways controlling neuronal migration. As we observed a reduced NK frequency upon GPR161 knockdown (Fig. 1H), we reasoned that GPR161 signaling might regulate cytoskeleton element targets known to be involved in the nuclear movement. In particular, the centrosome, closely linked to the PC, acts as a microtubule organization center extending a microtubule cage around the nucleus, necessary for NK (*23*–*26*). We thus monitored the organization of the microtubule cage upon GPR161 knockdown. To label the microtubule cage, we coelectroporated with the microRNAs a plasmid expressing doublecortin fused to red fluorescent protein (DCX-RFP) (*27*), which was detected by immunofluorescence on fixed brain sections.

**Fig. 3.**
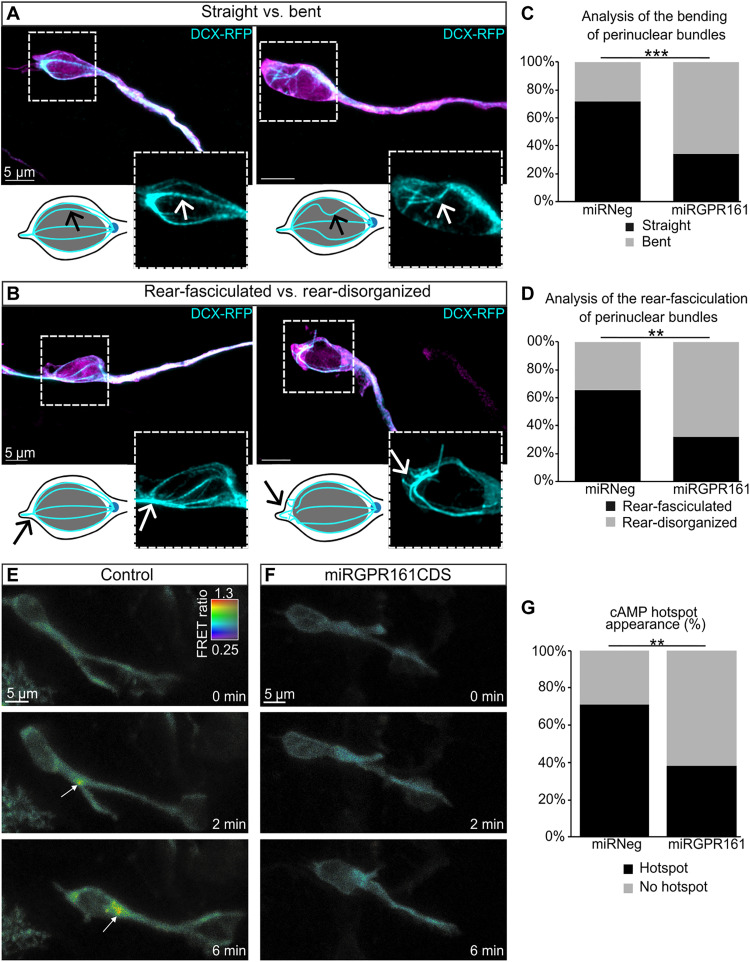
The ciliary receptor GPR161 regulates the organization of the microtubular nuclear cage and is the upstream receptor of cAMP signaling at the centrosome. (**A** and **B**) Immunohistochemistry of migrating neuroblasts in the RMS electroporated with either miRNeg or miRGPR161CDS. GFP immunolabeling (magenta) marks electroporated cells. DCX-RFP (cyan) labels the microtubular nuclear cage. Scale bars, 5 μm. (A) Illustrations of migrating neuroblasts presenting in the left a well-defined straight mictrotubular cage (from a miRNeg electroporated neuroblast) compared to the right, a bent microtubular cage (from a miRGPR161CDS electroporated neuroblast). (B) Illustrations of migrating neuroblasts presenting a well-defined rear-fasciculated microtubular cage in the left (from a miRNeg electroporated neuroblast) compared to a rear-disorganized microtubular cage in the right (from a miRGPR161CDS electroporated neuroblast). (**C** and **D**) Analysis of the percentage of the different microtubular cage features in miRNeg (*N* = 3, *n* = 46) and miRGPR161CDS (*N* = 5, *n* = 61) conditions. (C) Analysis of the straight versus bent microtubular cages. Pearson’s χ^2^ test (1, *N* = 107) = 14.606, *P* < 0.001. (D) Analysis of the rear-fasciculated versus rear-disorganized microtubular cages. Pearson’s χ^2^ test (1, *N* = 60) = 6.637, *P* < 0.01. (**E** and **F**) Live two-photon imaging of representative control (E) and miRGPR161CDS (F) neuroblasts coelectroporated with Epac-Sh187 cAMP biosensor. Scale bars, 5 μm. (E) White arrow shows a dynamic cAMP hotspot present during NK in the control condition. (F) The hotspot is not detected in the miRGPR161CDS condition. (**G**) Percentage of migrating neuroblasts with or without the presence of a cAMP hotspot in control (*N* = 9, *n* = 52) and miRGPR161CDS conditions (*N* = 6, *n* = 34). Pearson’s χ^2^ test with Yate’s continuity correction (1, *N* = 86) = 7.8509, *P* = 0.005. *P < 0.05, **P < 0.01, ***P < 0.001.

Qualitative differences in the organization of the cage of microtubules encircling the nucleus led us to quantify and compare two selected features of this cage in both conditions. First, we distinguished between cages formed exclusively by straight microtubule bundles from those containing bent microtubule bundles (Fig. 3A). Second, we considered the aspect of microtubule bundles at the rear of the nucleus, distinguishing rear-fasciculated versus rear-disorganized bundles (Fig. 3B). Our quantitative analyses show that while a great majority of miRNeg electroporated neuroblasts display cages with straight bundles of microtubules (71.73% straight and 28.27% bent), most miRGPR161CDS electroporated neuroblasts display bent bundles of microtubules (34.42% straight and 65.58% bent) (Fig. 3C). A similar inversion in the proportion of rear-fasciculated versus rear-disorganized was observed between miRNeg and miRGPR161CDS electroporated neuroblasts (65.52% rear-fasciculated and 34.48% rear-disorganized in miRNeg; 32.26% rear-fasciculated and 67.74% rear-disorganized in miRGPR161CDS) (Fig. 3D). These results demonstrate that the knockdown of GPR161 in migrating neuroblasts significantly perturbs the organization of their perinuclear cage of microtubules.

### GPR161 activates the PC-dependent cAMP signaling leading to a cyclic cAMP hotspot at the centrosome

GPR161 is a GPCR coupled to Gαs, hence able to trigger the production of cAMP through Gs-dependent activation of an adenylate cyclase (*16*). We recently reported the cyclic appearance of a cAMP hotspot at the centrosome of migrating neuroblasts during CK and NK (*11*). We further showed that ablation of the PC or knockdown of the ciliary adenylate cyclase 3 (AC3) prevented the production of this cAMP hotspot at the centrosome and led to migration defects including decreased NK frequency, increased pausing time, and decreased migration speed (*11*). This migratory phenotype was identical to the one induced by GPR161 knockdown (Fig. 1, H to J). This suggested that GPR161 could be upstream of this previously described cyclic accumulation of cAMP.

To test this hypothesis, we coelectroporated either the miRNeg or the miRGPR161CDS with a Förster resonance energy transfer (FRET) cAMP-specific biosensor (*11*) in postnatal mice and imaged cAMP signal in brain slices (Fig. 3F). While 71.2% of control cells displayed a cAMP hotspot (Fig. 3, E and G, and movie S13), only 38.2% of miRGPR161CDS displayed a cAMP hotspot (Fig. 3, F and G, and movie S14). The temporal correlation between the hotspot presence and NK, presented in fig. S4, does not show obvious difference in the temporal distribution of the hotspot with respect to NK. This indicates that GPR161 knockdown abolishes the accumulation of cAMP at the base of the cilium in a majority of migrating neuroblasts. Overall, ciliary GPR161 is the GPCR upstream of the cAMP signaling leading to a cyclic appearance of a cAMP hotspot at the centrosome.

### GPR161 signaling pathway regulates migration through cAMP/PKA-dependent phosphorylation of NDE1

We next asked how the GPR161-dependent centrosomal cAMP accumulation could influence the integrity of the microtubule nuclear cage. One target of cAMP at the centrosome is PKA. We previously showed that delocalization of PKA from the centrosome phenocopies the defects induced by PC deletion or AC3 knockdown (*11*), indicating that centrosomal cAMP/PKA could be key for NK. One centrosomal target of PKA is nudE neurodevelopment protein 1 (NDE1) (*28*). Unphosphorylated NDE1 belongs to a multiprotein complex containing lissencephaly-1 (LIS1) and dynein (*29*), two proteins crucial for the microtubule-dependent NK (*30*). This complex plays a key role in neuronal migration (*31*–*33*).

**Fig. 4.**
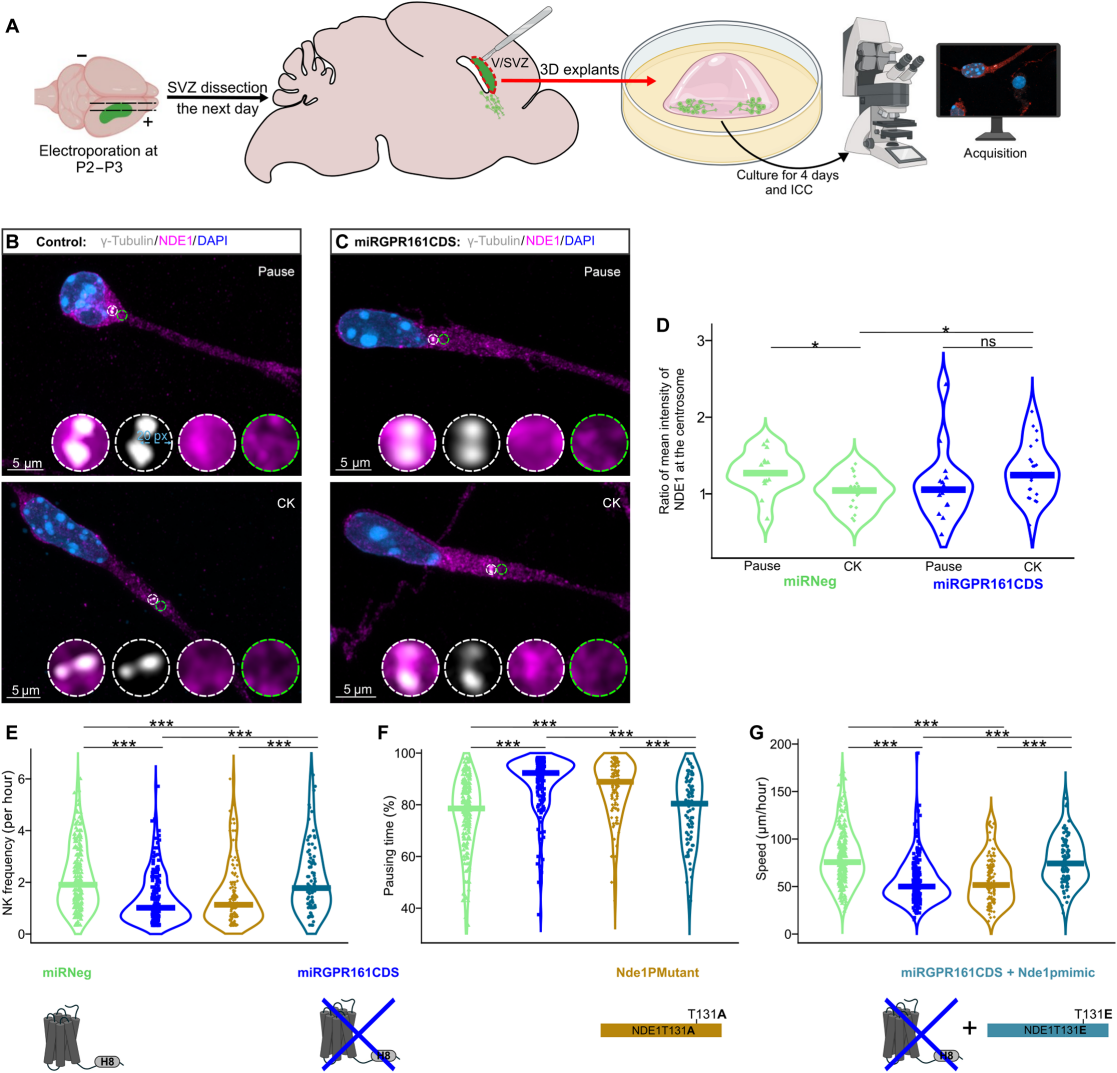
NDE1 is a downstream target of the cAMP/PKA signaling at the centrosome. (**A**) Experimental procedure of 3D explants. ICC, immunocytochemistry. (**B** and **C**) Illustrations of a control miRNeg (B) and miRGPR161CDS (C) electroporated neuroblast during pausing time (pause, top) and during CK phase (CK, bottom), showing NDE1 subcellular localization (magenta) and its concentration at the centrosome labeled by γ-tubulin (gray) and nuclear 4′,6-diamidino-2-phenylindole (DAPI) revelation in blue. The measurement of NDE1 mean intensity at the centrosome was performed within a circle of 20–pixel (px) radius, centered on the centrosome (white circle), and normalized with a same size circle adjacent in the leading process direction (green circle). (**D**) Analysis of the ratio of the mean intensities of NDE1 at the centrosome level in miRNeg (*N* = 3) and miRGPR161CDS (*N* = 4) neuroblasts, during pausing time (nucleus-to-centrosome distance: N-Ce < 6 μm; miRNeg: *n* = 15, miRGPR161CDS: *n* = 14) and CK phase (N-Ce ≥ 6 μm; miRNeg: *n* = 21, miRGPR161CDS: *n* = 23). Two-way analysis of variance (ANOVA): genotype effect (*F*_1,69_ = 1.203, *P* = 0.276), CK effect (*F*_1,69_ = 0.298, *P* = 0.586), and genotype CK interaction (*F*_1,69_ = 7.229, *P* = 0.009) followed by a Benjamini-Hochberg post hoc test. ns, not significant. (**E** to **G**) Rhythm of migration (see also fig. S5): (E) NK frequency per hour, (F) percentage of neurons in pausing time, and (G) speed of migration (micrometers per hour), in neuroblasts electroporated with miRNeg (green), miRGPR161CDS (dark blue), Nde1PMutant (brown), or miRGPR161CDS + Nde1pmimic (light blue). *P < 0.05, **P < 0.01, ***P < 0.001.

To determine whether NDE1 may be involved in the GPR161 pathway–regulating migration, we first analyzed the localization of NDE1, with a special focus on the centrosome. The high density of migrating neuroblasts in the RMS prevented the accurate characterization of NDE1 immunolocalization in brain tissue sections. We thus developed an ex vivo culture system, in which V/SVZ explants from postnatal electroporated mice were dissected and cultured in Matrigel for 4 days as previously described (*34*), followed by γ-tubulin (as a marker of the centrosome) and NDE1 immunodetection (Fig. 4A).

We determined the ratio of NDE1 mean intensity at the γ-tubulin–labeled centrosome to that in the cytoplasm adjacent to the centrosome [see white and green circles in Fig. 4 (B and C)]. In control neuroblasts (miRNeg) during the pausing phase (nucleus-to-centrosome distance, <6 μm), NDE1 labeling was enriched at the centrosome (most cells had a ratio > 1, mean ratio: 1.3 ± 0.3) (Fig. 4, B and D). However, during CK (nucleus to centrosome distance superior or equal to 6 μm), NDE1 labeling was more homogeneous throughout the cytoplasm, with no enrichment at the centrosome (ratio, ~1; mean ratio: 1.0 ± 0.3) (Fig. 4, C and D). This indicates that in control neuroblasts, the centrosomal enrichment of NDE1 during the pause phase is lost during CK (Fig. 4D). In GPR161-knockdown neuroblasts, the NDE1 ratio at the centrosome versus the adjacent cytoplasm was more variable compared to controls and, notably, showed no significant difference between the pause and CK phases (mean ratio during pause: 1.05 ± 0.4; mean ratio during CK: 1.24 ± 0.4) (Fig. 4, C and D). Most miRGPR161CDS neuroblasts in CK exhibited centrosomal enrichment of NDE1 compared to control miRNeg neuroblasts in CK (mean ratio, 1.27; *P* < 0.05) (Fig. 4, C and D). Together, these data suggest that activating the GPR161 pathway during migration influences NDE1 localization at the centrosome.

The perturbed centrosomal localization of NDE1 in GPR161 knockdown condition (Fig. 4, C and D) prompted us to interrogate whether PKA-dependent phosphorylation of NDE1 is involved in the GPR161-dependent regulation of migration. PKA phosphorylates NDE1 at threonine-131, which decreases its interaction with LIS1 (*28*). To test whether NDE1 phosphorylation is involved in neuronal migration, we analyzed in brain slices the effect on neuronal migration of a mutated form of NDE1 which cannot be phosphorylated by PKA [Nde1PMutant, with an alanine substitution of the threonine at position 131; fig. S1D and (*28*)] (Fig. 3, E to G, and movie S15). Notably, NDE1PMutant induced the same migratory defects as observed upon GPR161 knockdown: reduced NK frequency [1.1 (1.2) NK/hour], increased pausing time [88.9% (14.5)], and reduced migration speed [51.6 (35.4) μm/hour] (Fig. 3, E to G). Last, we tested whether a constitutive phospho-like state of NDE1 could rescue GPR161 knockdown in which NDE1 is delocalized. For that, we coelectroporated the miRGPR161CDS plasmid with a phosphomimetic form of NDE1 [Nde1pmimic, with a glutamate substitution of threonine-131; fig. S1D and (*28*)]. In this condition, all the migration parameters were rescued [NK frequency: 1.8 (1.8) NK/hour; pausing time: 80.2% (21.6); speed: 74.3 (35.4) μm/hour] (Fig. 4, E to G, and movie S16).

**Fig. 5.**
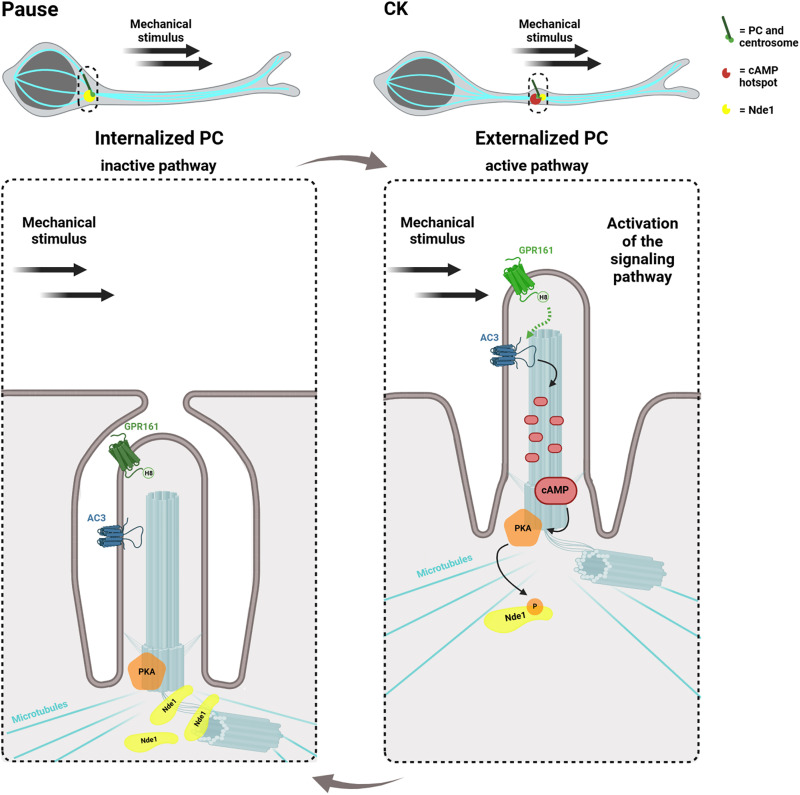
Scheme of cAMP/PKA/NDE1 pathway activation dependent on GPR161’s mechanosensitivity at the PC during CK. Migrating neuroblasts alternate between phases of pause and CK. During pausing time (pause), the PC is internalized, and the pathway remains in an inactive state. During CK, the PC, attached to the centrosome moves into a proximal leading process in the direction of migration, is externalized. The PC externalization allows the mechanosensitive receptor GPR161 to be exposed to the surface of the neuroblast and thus to mechanical stimuli. Mechanical activation of GPR161 through its helix 8 triggers the activation of the ciliary AC3, which in turn produces a cAMP hotspot at the centrosome. This cAMP hotspot activates PKA at the centrosome, enabling the phosphorylation of NDE1 at its threonine-131 residue. Activation of the pathway leads to a reduction in NDE1 localization at the centrosome. Created in BioRender. Allam, A. (2025) https://BioRender.com/2ff661a.

Collectively, these findings indicate that GPR161 and NDE1 phosphorylation at threonine-131 is part of the cAMP/PKA signaling pathway that regulates neuronal migration. On the basis of these findings, we propose a pathway initiated by the ciliary GPR161, which can act as a mechanosensor, as the PC is externalized at the onset of CK. This activation triggers AC3 activation via Gαs, resulting in the generation of a cAMP hotspot at the centrosome, which leads to NDE1 phosphorylation by PKA, regulating the organization of the microtubular nuclear cage (Fig. 5).

## DISCUSSION

The PC has emerged as a key mechanosensory organelle, capable of detecting and transducing mechanical signals essential for key developmental processes like the development of left-right asymmetry (*13*, *14*). Our study demonstrates that the mechanoreceptor GPR161 at the PC regulates neuronal migration. Notably, a previous study showed that knocking out GPR161 results in periventricular heterotopia and polymicrogyria (*35*), suggesting that the absence of GPR161 disrupts neuronal migration. We demonstrate here that the mechanosensory property of GPR161 plays a crucial role in neuronal migration. Fluid shear stress promotes NK in 2D-cultured migrating neuroblasts, and knockdown of GPR161 abolishes this flow-induced migration. Furthermore, our results reveal that the helix 8 domain of GPR161, linked to GPCR mechanosensitivity (*21*), is critical for its regulatory function on migration, since the GPR161H8del mutant fails to rescue migration defects. The GPR161 mechanosensitivity at the PC thus drives neuronal saltatory migration. In vitro, fluid shear stress activates neuronal migration through mechanical forces on GPR161, localized at the PC which cycles between externalization and internalization.

We did not formally identify the mechanical stimulus acting on the PC GPR161 in vivo. We cannot exclude the possibility that interstitial fluid flow, previously estimated to be between 0.076 and 0.53 Pa in rodent brain tissue (*36*), may play this role. However, the extremely high density of migrating neuroblasts in the postnatal RMS, providing very little extracellular space for interstitial fluid flow (*37*, *38*), does not sustain this hypothesis. Equally unlikely is the hypothesis that intrinsic morphological changes of neuroblasts contributing to mechanical deformation of the cilium are solely responsible for mechanosensitive regulation. Should it be the case, the migration of neuroblasts in 2D cultures should remain optimal in the no flow condition. As an alternative hypothesis, we propose that the PC-dependent regulation of migration is driven by frictional forces generated by cell-cell interactions. It has been shown that the immediate extracellular environment of the externalized primary cilia in migrating neuroblasts in the RMS are the surrounding cells that are used as substrate for their migration, primarily other migrating neuroblasts (*10*). The dynamic behavior of the cilium likely modulates the mechanical stimulus on it, which in turn would impose a rhythmic saltatory movement of neuroblasts during migration.

The mechanisms controlling the externalization and internalization of the PC have never been addressed in neuroblasts. In epithelial and fibroblast cells, subdistal appendages (sDAP)—sheet-like structures that facilitate centriole docking to the plasma membrane—play a key role in maintaining the PC internalized (*39*). Mazo *et al.* (*40*) further showed that disruption of sDAP results in externalization of the PC to the cell surface, which can potentially alter both mechanical and chemical signal transduction. Further studies are needed to determine whether similar mechanisms are at play upon saltatory migration of neuroblasts.

Our data identify GPR161 as the upstream receptor of the cAMP/PKA-ciliary pathway previously described (*11*), as its knockdown leads to the disappearance of the cAMP hotspot during neuronal migration. GPR161 was initially described as a basal repressor of the Hedgehog pathway via cAMP signaling (*16*), and previous studies have shown an implication of Hedgehog signaling in neuronal migration (*6*, *41*). Nevertheless, our results strongly suggest that the GPR161 function described here does not involve Hedgehog signaling. No Sonic Hedgehog-expressing cells were detected in the adult RMS (*42*). Furthermore, canonical Hedgehog signaling activation typically delocalizes GPR161 from the cilium (*16*). In contrast, we observed a consistent presence of GPR161 at the PC in migrating neuroblasts. Our findings thus reveal a previously unknown role for GPR161 upstream of the cAMP/PKA signaling pathway, aligning with recent studies that suggest a broader function for GPR161 beyond Hedgehog signaling, notably in promoting tumor cell invasion in triple-negative breast cancer (*20*, *43*, *44*).

We also demonstrate that NDE1 is a downstream target of the pathway. First, knockdown of GPR161 disrupts the cAMP-ciliary signaling pathway and abolishes the difference observed under control conditions, where NDE1 is enriched at the centrosome during the pausing phase but not during NK (Fig. 4). Then, NDE1 has been shown to be phosphorylated at the centrosome by PKA on its threonine-131 (T131) (*28*). Noteworthy, our data showed that the overexpression of a nonphosphorylated form of NDE1 on its T131 leads to migration defects similar to those observed in AC3 knockdown or PKA delocalization from the centrosome (*11*). In contrast, overexpression of a phosphorylated-like form of NDE1 on its T131 rescues all migration parameters in GPR161 knockdown neuroblasts. These findings are in line with prior studies showing that NDE1 loss of function leads to human lissencephaly (*45*, *46*) and schizophrenia (*33*), further supporting the relevance of this protein in proper neuronal migration and development.

Together, our findings highlight a pathway initiated by GPR161 at the PC, potentially activated via its helix 8 by a mechanical stimulus from the external environment as the PC emerges at the onset of neuroblast migrating phase. This activation leads to the activation of AC3 via Gαs, resulting in the generation of a localized cAMP hotspot at the centrosome. This cAMP signal activates centrosomal PKA, which phosphorylates NDE1 on its T131 (*28*). It is known that NDE1 forms a complex with Lis1 (*31*, *32*) and that the phosphorylation of NDE1 on its T131 by PKA leads to a decreased association with Lis1 (*28*). Our data suggest that upon activation of the pathway, phosphorylated NDE1 may be released from the centrosome. The dissociation of the complex may allow Lis1 to carry out functions in other cellular compartments, such as at the microtubular cage surrounding the nucleus, where it enhances the processivity of dynein on microtubules (*31*, *32*). Such a hypothesis is consistent with previous studies that identified the Lis1/dynein complex at the nuclear cage (*47*) and demonstrated that knockdown of Lis1 or dynein disrupts the microtubule network around the nucleus in cortical neuron cultures (*48*).

To conclude, our study identifies GPR161 as a mechanoreceptor at the PC, regulating neuronal migration via its helix 8 domain. This mechanism is likely general, as GPR161 regulates the cAMP-ciliary pathway previously shown to regulate diverse forms of neuronal migration (*11*). We also establish NDE1 as a critical downstream effector, as it is phosphorylated by PKA at the centrosome. Our findings reveal a cilium-based dynamic pathway that integrates rhythmic mechanosensitivity to drive neuronal saltatory migration (Fig. 5). Dysregulation of this rhythm, potentially due to impaired mechanosensation, could delay neuronal arrival and integration, ultimately leading to malformations in neuronal network development and altered functioning. These results thus open avenues for deciphering the role of mechanics and cilia in normal and pathological development.

## MATERIALS AND METHODS

### Mice

E14 pregnant C57BL6-J mice were purchased from Janvier Labs and housed in a 12-hour light/dark cycle, in cages containing two females. The postnatal mice were housed in the cages with their mother. Animal care was conducted in accordance with standard ethical guidelines [National Institutes of Health (NIH) publication no. 85-23, revised 1985 and European Committee Guidelines on the Care and Use of Laboratory Animals 86/609/EEC]. The experiments were approved by the ethic committee (Comité d’Ethique en Expérimentation Animale Charles Darwin C2EA-05 and the French Ministère de l’Education Nationale de l’Enseignement Supérieur et de la Recherche, projects APAFIS#1364-2018021915046521 and APAFIS# #45297-2023102517215274). We strictly performed the approved procedures.

### Plasmids and viruses

#### Plasmids

Silencing of GPR161 has been performed using BLOCK-iTTM Pol II miR RNA interference (RNAi) Expression Vector kits (Invitrogen) and the RNAi Designer (Invitrogen). All the plasmids were used at concentrations between 4 and 8 μg/μl (0.01% Fast green) for postnatal electroporation. When several plasmids were coinjected, the ratio of each was calculated to reach equimolarity to transduce cells. The sequence of the single-stranded oligonucleotides for miRGPR161CDS are as follows: top: TGCTGAATACAGCCAAGAGAGTGTGTGTTTTGGCCACTGACTGACACACACTCTTGGCTGTAT; bottom:CCTGAATACAGCCAAGAGTGTGTGTCAGTCAGTGGCCAAAACACACACTCTCTTGGCTGTATTC.

The double-stranded oligos were inserted in a pcDNATM6.2-GW/EmGFP-miR (EmGFP: Emerald Green Fluorescent Protein). To produce the pcDNATM6.2-GW/Tdtomato-miR, EmGFP was replaced by TdTomato using the Dra1 restriction enzyme. The resulting constructions were sequenced and validated before use. The sequence of the single-stranded oligonucleotides for miRGPR161UTR are as follows: top: TGCTGTACAGAAGACAACTGAAGTCAGTTTTGGCCACTGACTGACTGACTTCATGTCTTCTGTA; bottom: CCTGTACAGAAGACATGAAGTCAGTCAGTCAGTGGCCAAAACTGACTTCAGTTGTCTTCTGTAC.

Constructs of pRP-CMV > mGpr161-wt and pRP-CMV > mGpr161-h8deleted were designed by VectorBuilder. For the pRP-CMV > mGpr161-h8deleted, only the amino acids NKTVRKELLGMC (from no. 344 to no. 355) representing the helix 8 were deleted. pNDE1T131A (Nde1PMutant) and pNDE1T131E (Nde1pmimic) were given by N. Bradshaw (*28*). pDCX-RFP (Doublecortin-Red Fluorescent Protein), initially produced by J. Gleeson, was obtained from Addgene (plasmid #32851; http://n2t.net/addgene:32851; RRID:Addgene_32851).

#### Viruses

The pLV-CMV > EGFP-miRGPR161 and pLV-CMV > EGFP-miRNeg viruses were designed by VectorBuilder. VSV-G pseudotyped third-generation lentivirus was used. The same sequences of the single-stranded oligos for miRGPR161 and miRNeg were used as in the plasmids above. When used in microfluidic experiments, viruses were added at a multiplicity of infection of 1 in the resuspension complete medium, before plating the cells.

### Postnatal electroporation

Postnatal electroporation was performed at P2, P3, or P4 in C57BL/6-J mouse strains. The postnatal mice were anesthetized by hypothermia. Pseudo-stereotaxic injection [from lambda, mediolateral (ML): −0.8, antero-posterior (A/P): 1.1, dorso-ventral (D/V): 2 at P2; ML: 1.5, A/P: 2, D/V: 2.5 at P3; and ML: 1.5, A/P: 2, D/V: 2.5 at P4] (using a glass micropipette, 50 μl Drummond Scientific Wiretrol I, 5-000-1050) was performed, and 2 μl of plasmid (between 3 and 10 μg/μl) was injected. Animals were subjected to five pulses of 99.99 V during 50 ms separated by 950 ms using the CUY21 SC electroporator and a 10-mm tweezer electrode (CUY650-10, Nepagene). The animals were placed on 37°C plates to restore their body temperature before returning with their mother. Animals were considered as fully restored when pups were moving naturally and their skin color returned to pink.

### Acute brain slices

Brain slices from mice aged from P6 to P10 were prepared as previously described (*11*). Briefly, pups were killed by decapitation, and the brain was quickly removed from the skull. Sagittal brain slices (250 μm) were cut with a VT1200S vibratome (Leica). Slices were prepared in the ice-cold cutting solution of the following composition: 125 mM NaCl, 0.4 mM CaCl_2_, 1 mM MgCl_2_, 1.25 mM NaH_2_PO_4_, 26 mM NaHCO_3_, 5 mM sodium pyruvate, 20 mM glucose, and 1 mM kynurenic acid, saturated with 5% CO_2_ and 95% O_2_. Slices were incubated in this solution for 30 min at room temperature and then placed in a recording solution (identical to the solution used for cutting, except that the Ca^2+^ concentration was 2 mM and kynurenic acid was absent) for at least 30 min at 32°C before image acquisition.

### Time-lapse video microscopy of migration

To analyze cell migration, images were obtained with an inverted SP5D confocal microscope (Leica) or an upright two-photon microscope Leica SP5 MPII. Images were taken every 3 min for 2 to 3 hours using a 40×/1.25 numerical aperture (NA) objective with 1.5x optical zoom on the inverted confocal microscope and with a 25×/0.95 NA objective, 1.86× optical zoom. The temperature in the microscope chamber was maintained at 32°C, for postnatal imaging, and brain slices were continuously perfused with a heated recording solution (see above) saturated with 5% CO_2_ and 95% O_2_.

Biosensor images were acquired with an upright two-photon microscope Leica SP5 MPII with a 25×/0.95 NA objective, 4× optical zoom, and a GaAsP hybrid detector. The excitation wavelength was set at 850 nm to excite the mTurquoise2 donor. The two emission wavelengths were acquired simultaneously with filters of 479+/−20 and 540+/−25 nm. Image stacks with 1-μm intervals were taken every 2 min for 1 hour. The presence of TdTomato, indicative of the presence of microRNA, was assessed with a confocal head. The temperature in the microscope chamber was maintained at 32°C, and brain slices were continuously perfused with a heated recording solution (see above) saturated with 5% CO_2_ and 95% O_2_.

### Microfluidic experiments

Pups were sacrificed by decapitation, and the brain was quickly removed from the skull. SVZ tissues were dissected in L-15 medium and incubated in 0.25% trypsin at 37°C for 5 min. Subsequently, cells were dissociated in deoxyribonuclease (DNase) solution [1 ml of L-15 + 500 μl of fetal bovine serum + 200 μl DNase (0.08 mg/ml)] and counted using a Malassez counting chamber. Cells were resuspended in complete medium.

Commercial Ibidi channels (μ-Slide VI 0.4) were coated with 10% Matrigel diluted in L-15 and incubated for 45 min at 37°C in a humidity-controlled incubator. After rinsing with complete medium, 200,000 cells in 65 μl of medium were added to each channel and placed in a 37°C, 5% CO_2_ incubator for overnight incubation.

Live imaging was performed the next day using a Zeiss inverted 20×/0.95 NA objective and 0.6 optical zoom. The temperature in the microscope chamber was maintained at 37°C with 5% CO_2_.

Flow experiments were conducted using a microfluidic flow system (Flow EZ345, Fluigent) with the pressure controller set to induce a fluid flow of medium inside a commercial microfluidic channel of known dimensions (μ-Slide VI 0.4, Ibidi). The flow rate was set constant with the controller, in the course of 2.5 hours. The pressure pumps were set to apply a shear stress τ of 0.136 Pa, calculated using the following equation: τ = $\frac{6\eta Q}{h^{2}w}$ , where η is the medium fluid viscosity (evaluated at 7.10^−4^ Pa·s), *Q* is the flow rate (consistently measured as 1.2 ml/min and controlled by the pressure pumps and limited by the known channel’s dimensions), *h* is the microchannel height (400 μm), and *w* its width (3.8 mm).

The interstitial fluid flow within rodent brain tissue is still unknown but was estimated to be between 0.076 and 0.53 Pa (*36*). We have decided to use the value of 0.13 Pa as a lower-intermediate amplitude. Experimentally, lower values than 0.13 Pa did not seem to affect our cell response, while a higher value detached cells. Control experiments without flow were conducted under the same conditions as above and with live imaging for 2.5 hours in the same microscopy system but without connecting the microfluidic pump.

### Analysis of neuronal migration

Analyses were performed using ImageJ (NIH Image; NIH, Bethesda, MD) software and MTrackJ plugin. The nucleus of each cell was tracked manually on each time frame during the whole movie. For cell migration, calculation of speed, pausing time, and nuclear translocation frequency were performed using the *x*, *y*, and *t* coordinates of the nucleus of each cell. Cells were excluded from the analysis if they were tracked during less than 30 min or did not perform any nuclear translocation during the whole tracking. A cell was considered as migrating if it performed a distance superior to 6 μm during a 3-min interval. More specifically, speed was calculated by summing all the distances traveled by one cell and dividing the total distance by the total time, including time slots (3-min interval) when the cell pauses. The pausing time is calculated as the percentage of time spent in pause, defined as the sum of the time slots during which the cell moves less than 6 μm, hence below the cutoff for an NK.

### Analysis of biosensor images

The Epac-SH187 cAMP biosensor is composed of a part of Epac protein coupled to a donor and an acceptor fluorophore. This biosensor displays a high ratio change and excellent photostability for measuring live cAMP concentration in the micromolar range. It switches from a high FRET conformation to a lower FRET conformation upon binding of cAMP. The maximum intensity was projected vertically to form a 2D image. Changes in cAMP concentration were analyzed with the plugin FRETratioFx on ImageJ to create a ratio image of non-FRET over FRET fluorescence intensity, which reports a biosensor cAMP activation level. The ratio for each pixel is calculated and converted into a hue value.

### Immunohistochemistry

P7 to P10 mice were deeply anesthetized with 0.1 ml of Euthasol (sodium pentobarbital at 40 mg/ml). Intracardiac perfusions with 4% paraformaldehyde (PFA) were performed. Brains were postfixed overnight in 4% PFA. Three rinses were done with 1× phosphate-buffered saline (PBS) (Gibco, 1400-067). Seventy-micrometer sagittal slices were cut with a VT1200S vibratome (Leica). Then, slices were placed for 1 hour in a saturation solution (10% fetal bovine serum; 0.5% Triton X-100 in PBS). The primary antibodies used in this study were as follows: chicken anti-GFP (1:2000; Aves, GFP-1020), rabbit anti-GPR161 (1:50; Proteintech, 13398-1-AP), mouse anti-Arl13b (1:1000; NeuroMab, N295B/66), mouse anti–γ-tubulin (1:500; Sigma-Aldrich, T6557), rabbit anti-NDE1 (1:100; Proteintech, 10233-1-AP), and rabbit anti-DsRed (1:500; Takara, 632496).

For GPR161 immunostaining, a pretreatment of antigen retrieval was done with an incubation of slices in 1 ml of 10 mM citrate buffer for 15 min at 95°C, followed by rinses with 1× PBS. The antibodies were diluted in the saturation solution. Slices were incubated for 48 hours at 4°C under agitation with the antibodies. Three rinses of 20 min were performed with 1× PBS.

The secondary antibodies used were as follows: anti-chicken immunoglobulin Y Alexa Fluor 488 (1:1000; Jackson ImmunoResearch, 703-545-155) against anti-GFP; anti-rabbit Alexa Fluor 594 (1:1000; Jackson ImmunoResearch, 711-587-003) against anti-GPR161 and anti-DsRed; anti-mouse immunoglobulin G (IgG), Fcg subclass 2A–specific Alexa Fluor 647 (1:1000; Jackson ImmunoResearch, 115-605-206) against anti-Arl13b; and anti-mouse IgG, Fcg subclass 1–specific Alexa Fluor 647 (1:1000; Jackson ImmunoResearch, 115-585-205) against anti–γ-tubulin.

The antibodies were diluted in saturation solution. Slices were incubated for 1 hour at room temperature under agitation with the secondary antibody solution. Three rinses with 1× PBS were done. Slices were counter-colored with Hoeschst 1:500 and mounted with Mowiol. Acquisitions were performed using a Zeiss LSM 980 upright microscope at high magnification (objective 63×, zoom 3).

To quantify GPR161 knockdown, an Arl13b-positive PC was considered GPR161 negative when it was clearly immuno-negative or indistinguishable from the background at high magnification (objective 63×, zoom 3). 3D reconstructions of migrating neuroblasts were performed using Imaris (Carl Zeiss).

### Immunostaining on 2D-cultured neuroblasts

For the analysis of 2D-cultured neuroblasts, such as those used in microfluidic experiments, same dissociation and coating protocol were used as described above (see the “Microfluidic experiments” section). Here, neuroblasts were cultured in 12-well removable slide chambers (Ibidi, 81201). After overnight incubation at 37°C, 5% CO_2_, cells were fixed in 2% PFA for 30 min and then rinsed three times with 1× PBS. Primary antibodies were incubated overnight in the saturation solution at 4°C under agitation. Three rinses (20 min each) were performed with 1× PBS. A secondary antibody was incubated for 1 hour at room temperature under agitation. Three rinses (20 min each) with 1× PBS were done. 2D cultures were counter-colored with Hoeschst 1:500 and mounted with Mowiol.

### Analysis of microtubular cages

To analyze the organization of the microtubular cage in migrating neuroblasts, pDCX-RFP was coelectroporated with either miRNeg-GFP or miRGPR161CDS-GFP at postnatal stages P2 and P3. Immunostaining was performed as described in the above “Immunohistochemistry” section. High-magnification images of electroporated neuroblasts were acquired using a 63× objective with a 3× zoom. Each neuroblast was qualitatively assessed within stacks and categorized on the basis of two criteria:

1) Analysis of the bending of the microtubular cage: All microtubule bundles are straight and follow the curvature of the nucleus, or at least one bundle deviates from this curvature and appears bent.

2) Analysis of the rear-fasciculation of microtubular cage: All microtubule bundles reaching the rear of the nucleus fasciculate at the rear of the nucleus, or at least one bundle fails to fasciculate and appears disorganized.

### Immunostaining on SVZ explants in Matrigel

For the immunostaining of NDE1, the SVZ of electroporated mice was dissected as described (*34*). SVZ explants were placed on glass-bottom culture dishes (MatTek Corporation, P35G-0-20-C) within 10 ml of 60% Matrigel (Corning, 356237). After Matrigel solidification (15 min at 37°C, 5% CO_2_), culture medium was added, and the dishes were incubated for 4 to 5 days at 37°C, 5% CO_2_. For NDE1 immunostaining, SVZ cultures were fixed in 2% PFA for 30 min and then rinsed three times with 1× PBS. Primary antibodies anti-NDE1 and anti–γ-tubulin were incubated during 4 days in the saturation solution at 4°C under agitation. Five rinses (1 hour each) were performed with 1× PBS. The secondary antibodies were incubated for 2 hours at room temperature under agitation. Three rinses (1 hour each) with 1× PBS were done. Explants were counter-colored with Hoechst 1:500 and mounted with Mowiol.

### Image analysis and semiquantitative analysis of NDE1 immunoreactivity

NDE1 immunostaining was acquired using a Zeiss LSM 980 upright at high magnification (objective 63×, zoom 3). A circle region of interest (ROI; 40 pixel diameter, corresponding to 1.5 μm) centered on centrosome (labeled by γ-tubulin) was manually defined in Fiji. As a reference, a second ROI (40 pixel diameter) was defined next to the centrosomal ROI in the direction of the leading process. Quantification of NDE1 immunofluorescence was performed on the centrosomal z-section, through calculation of the ratio between the centrosomal ROI mean intensity and the reference ROI mean intensity, and the data were exported as Comma Separated Values (CSV) files for further analysis.

### Statistical analysis

All data manipulation and statistical analyses were performed using R (version 4.3.2, R Foundation for Statistical Computing, Vienna, Austria). Normality of variable distributions was assessed with the Shapiro-Wilk test, while the Levene test was used to evaluate the homogeneity of variances across groups. For variables that failed the Shapiro-Wilk or Levene tests, nonparametric methods were applied, including the one-way Kruskal-Wallis analysis of variance (ANOVA) on ranks, followed by the by Dunn’s post hoc test with Benjamini-Hochberg *P* value correction or Mann-Whitney rank sum tests for pairwise comparisons. Variables that met normality assumptions were analyzed using one-way ANOVA with Benjamini-Hochberg corrections for multiple comparisons or Student’s *t* test for two-group comparisons. Full statistical analysis of rhythm of migration parameters are shown in fig. S5.

As the variables of interest presented in table S1 were categorical at the single-cell level, individual cells were used as the unit of analysis in the corresponding experiments. We confirmed that there were no significant differences between animals within each condition (table S1), supporting this approach.

The orientation of cell migration trajectories, treated as circular variables, was analyzed between groups using circular ANOVA based on the likelihood ratio test. Categorical variables were compared using Pearson’s χ^2^ test or Fisher’s exact test. A *P* value of <0.05 was considered the threshold for statistical significance. Statistical significance: *P < 0.05, **P < 0.01, ***P < 0.001. Results are expressed as the median (interquartile range), with detailed descriptions of statistical tests provided in each figure legend.
