# Supplementary material for: GPR161 mechanosensitivity at the primary cilium drives neuronal saltatory migration
Source: Sci Adv. 2025 Jul 30;11(31):eadx3846. doi: 10.1126/sciadv.adx3846 (PMC12309678; doi:10.1126/sciadv.adx3846)
Supplement: Supplementary file 1 — Figs. S1 to S5 Table S1 Legends for movies S1 to S16 [file sciadv.adx3846_sm.pdf]

Science Advances
AAAS

Supplementary Materials for

GPR161 mechanosensitivity at the primary cilium drives neuronal saltatory migration

Théo Paillard *et al.*

Corresponding author: Alain Trembleau, alain.trembleau@sorbonne-universite.fr

Sci. Adv. **11**, eadx3846 (2025)
DOI: 10.1126/sciadv.adx3846

The PDF file includes:

Figs. S1 to S5
Table S1
Legends for movies S1 to S16

Other Supplementary Material for this manuscript includes the following:

Movies S1 to S16

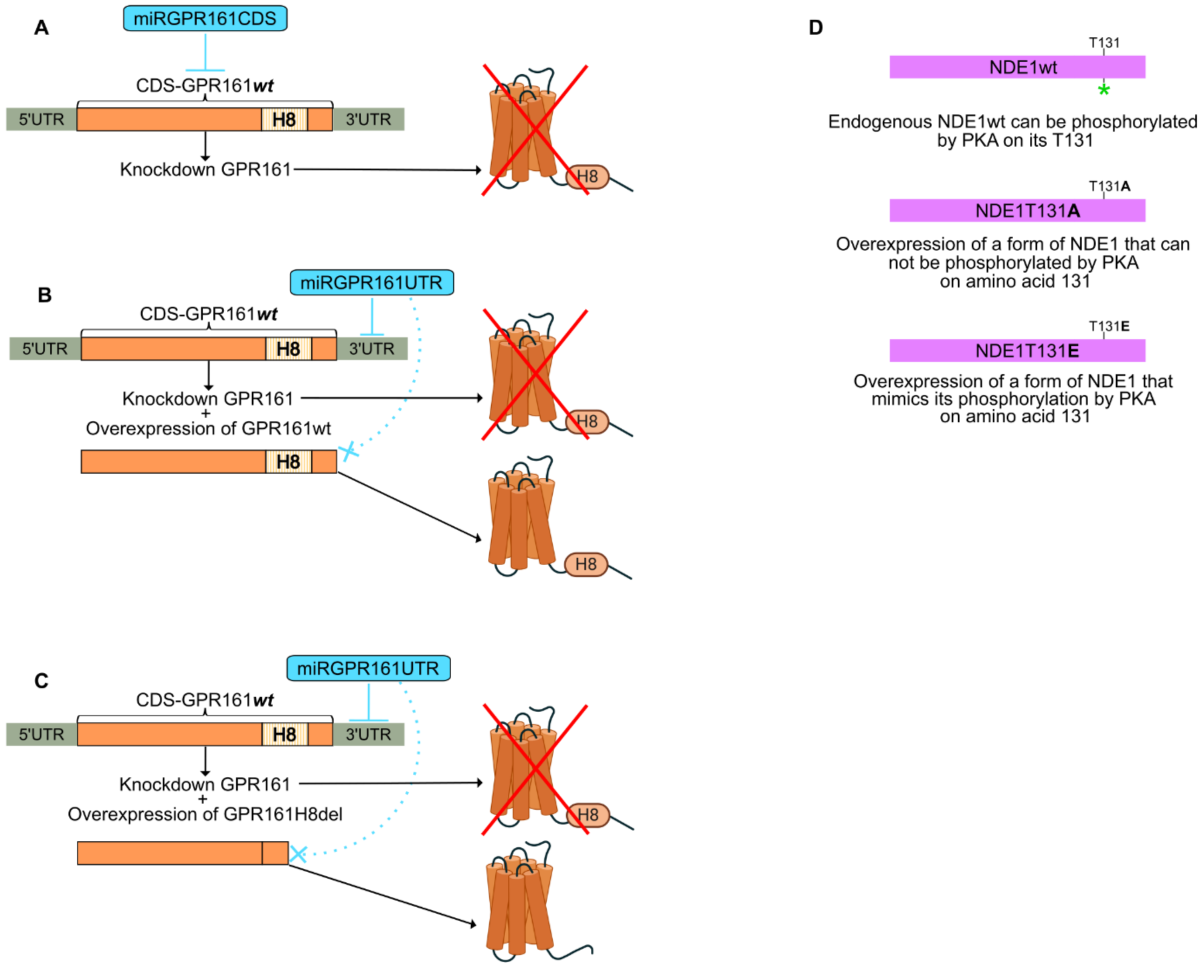

Fig. S1. Overview of the constructs used in this study.

(**A**) The miRGPR161CDS plasmid targets the coding sequence (CDS) of endogenous GPR161, leading to its knockdown. (**B**) The miRGPR161UTR plasmid targets the 3′ untranslated region (3′UTR) of endogenous GPR161, also resulting in its knockdown. The plasmid expressing wild-type GPR161 (GPR161wt) is overexpressed and cannot be targeted by miRGPR161UTR due to the absence of a 3′UTR, thereby rescuing GPR161wt expression. (**C**) Similar to (B), the plasmid miRGPR161UTR knocks down endogenous GPR161 by targeting its 3′UTR. Meanwhile, a mutant version of GPR161 lacking Helix 8 (GPR161H8del) is overexpressed. As this mutant also lacks the 3′UTR, it is not targeted by miRGPR161UTR, enabling the expression of the mutant form of GPR161. (**D**) Endogenous NDE1 can be phosphorylated (green asterisk) at the centrosome by PKA on Threonine 131 (T131) (top panel). In the NDE1PMutant condition, we overexpress a non-phosphorylatable NDE1 mutant, where T131 is replaced with alanine (middle panel). In the NDE1PMimic condition, we overexpress a phosphomimetic form of NDE1, where T131 is replaced with glutamic acid to mimic phosphorylation by PKA (bottom panel).

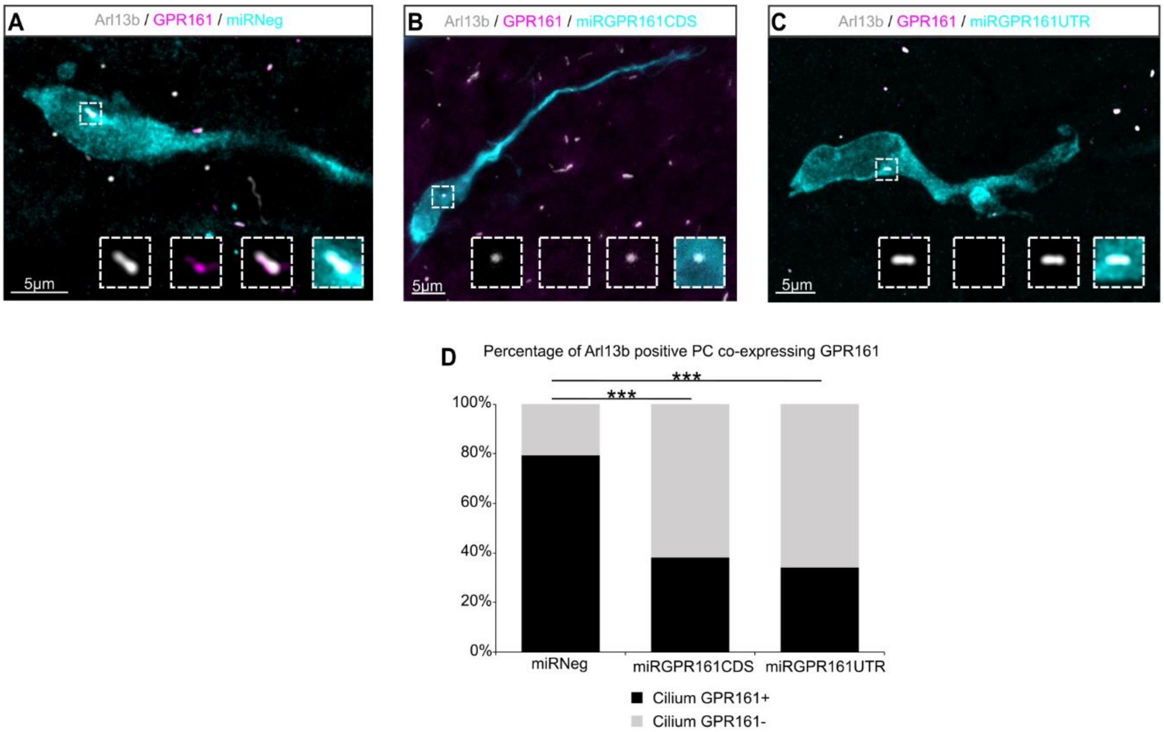

Fig. S2. Efficiency of miRGPR161CDS and miRGPR161UTR knockdowns.
(A-C) Immunohistochemistry experiments of neuroblasts electroporated with (**A**) miRNeg (**B**) miRGPR161CDS or (**C**) miRGPR161UTR plasmid. (**A**) The miRNeg electroporated neuroblast displays an intact Arl13b immunopositive PC (grey) with GPR161 immunoreactivity (magenta). (**B-C**) Both miRGPR161CDS and miRGPR161UTR display an intact Arl13b immunopositive PC (grey) but without GPR161 immunoreactivity (magenta). Scale bars: 5µm. (**D**) The percentage of electroporated neuroblasts displaying an Arl13b and GPR161 immunoreactive PC is significantly reduced in miRGPR161CDS (38.3%; N=3 n=60) and miRGPR161UTR (34.2%; N=3 n=38) electroporated neuroblasts compared to the control miRNeg (79.3%; N=3 n=53). Pearson's X2 test (2, N = 151) = 25.05, p <0.001.

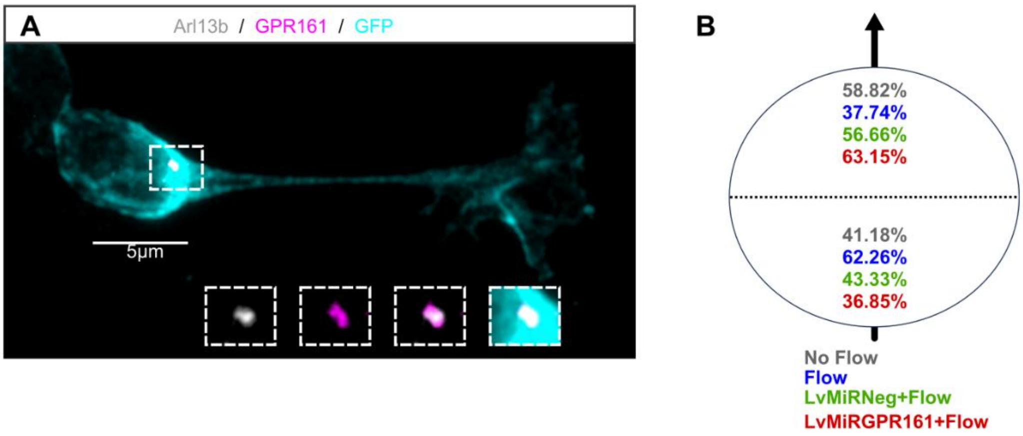

Fig. S3. Characterization of the 2D culture system and analysis of migration directionality in the microfluidics experiments.

(**A**) Immunohistochemistry of a 2D-cultured neuroblast (GFP+ cyan) showing GPR161 immunoreactive subcellular expression (magenta) in the Arl13b-positive PC (gray). Scale bar: 5 µm. (**B**) Migration directionality radar represented in 2 spatial dials. Percentage of cells migrating in either spatial direction, relative to the direction of the flow (arrow) in the different conditions: grey: no flow; blue: flow; green: LvMiRNeg+flow; red: LvMiRGPR161+flow. Circular analysis of variance based on the likelihood ratio test: $p = 0.60$.

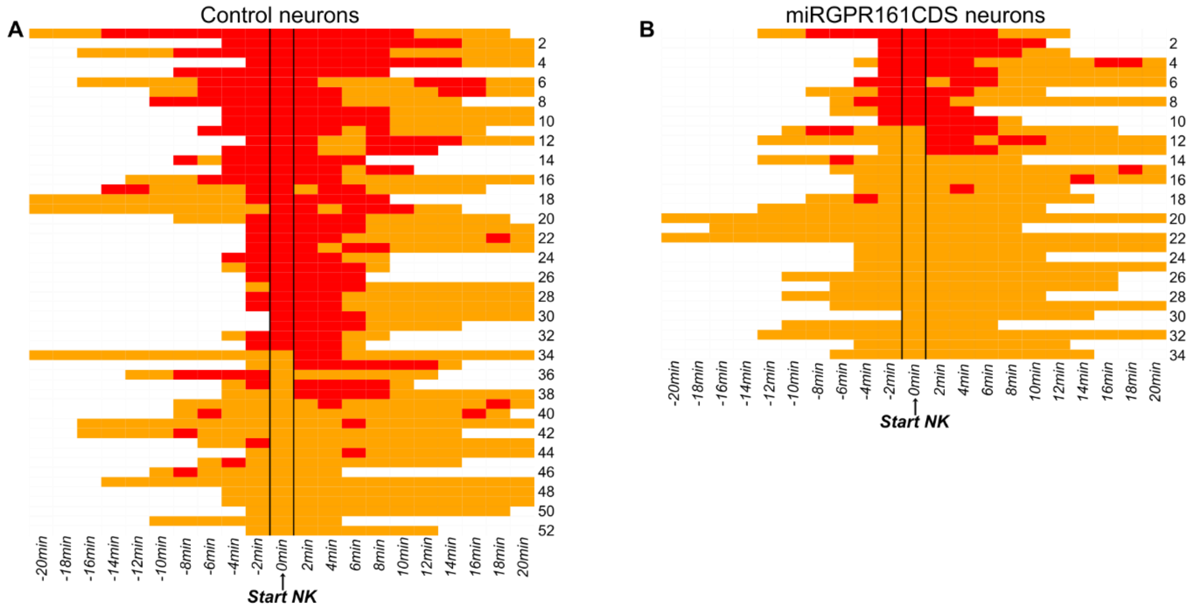

Fig. S4: Timing of the cAMP hotspot occurrence.
(**A-B**) Heatmaps showing the temporal dynamics of hotspot presence in migrating (**A**) control neurons or (**B**) mutant miRGPR161CDS neurons. Time 0 is defined as the start of NK, and visually marked between the two black lines. Each row represents a single cell, and each column represents a time point with two-minutes interval. The presence of the hotspot at a given time point is represented as a red rectangle and its absence as an orange rectangle. White rectangle corresponds to nonrecorded time points. The frequency of the hotspot presence (red) is reduced in miRGPR161CDS electroporated neurons as compared to control ones.

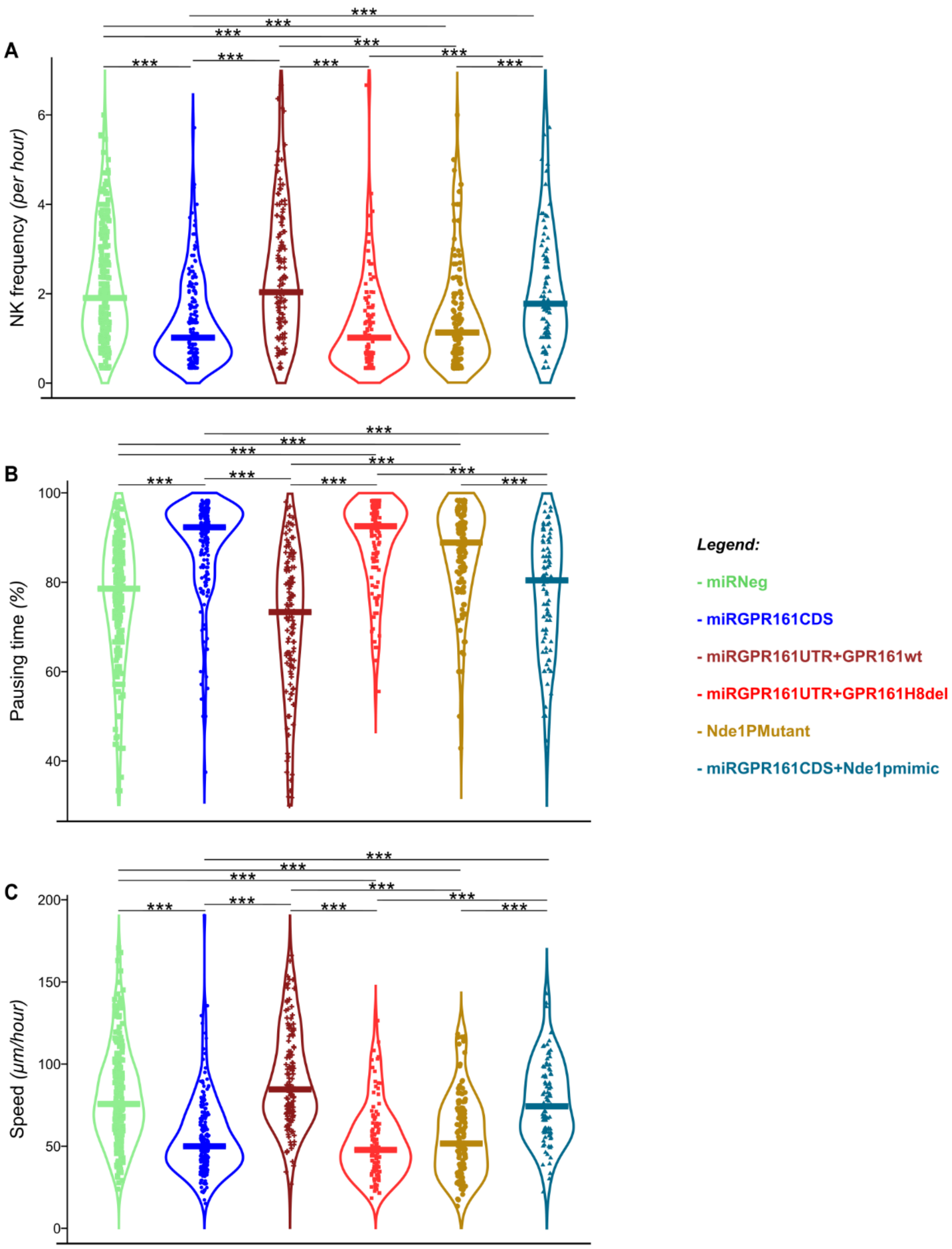

Fig. S5. Statistical analysis of the rhythm of migration.

(**A-C**) Migration parameters of neuroblasts electroporated with miRNeg (N = 6, n = 227), miRGPR161CDS (N = 7, n = 203), miRGPR161UTR+GPR161wt (N = 3, n = 139), miRGPR161UTR+GPR161H8del (N = 3, n = 114), Nde1PMutant (N = 4, n = 128) or miRGPR161CDS+Nde1pmimic (N = 3, n = 88) in C57/Bl6 background mice. (**A**) Analysis of NK frequency: miRNeg: 1.9 [1.8] NK/hour; miRGPR161CDS: 1.0 [1.1] NK/hour; miRGPR161UTR+GPR161wt: 2.0 [2.0] NK/hour; miRGPR161UTR+GPR161H8del: 1.0 [1.0] NK/hour; Nde1PMutant: 1.1 [1.2] NK/hour; miRGPR161CDS+Nde1pmimic: 1.8 [1.8] NK/hour. Kruskall-Wallis Test (Chi2 = 130.46, p-value<0.001, df = 2; followed by Dunn's posthoc test with Benjamini-Hochberg p-value correction). (**B**) Analysis of pausing time: miRNeg: 78.6% [19.3%]; miRGPR161CDS: 92.3% [10.2%]; miRGPR161UTR+GPR161wt: 73.3% [21.7%]; miRGPR161UTR+GPR161H8del: 92.5% [12.0%]; Nde1PMutant: 88.9%

[14.5%]; miRGPR161CDS+Nde1pmimic: 80.2% [21.6%]. Kruskall-Wallis Test (Chi2 = 242.98, p-value <0.001, df = 2; followed by Dunn's posthoc test with Benjamini-Hochberg p-value correction). (**C**) Analysis of speed of migration miRNeg: 75.7 [36.6] µm/hour; miRGPR161CDS: 50.0 [26.9] µm/hour; miRGPR161UTR+GPR161wt: 84.6 [39.0] µm/hour; miRGPR161UTR+GPR161H8del: 47.8 [27.0] µm/hour; Nde1PMutant: 51.6 [35.4] µm/hour; miRGPR161CDS+Nde1pmimic: 74.3 [35.4] µm/hour. Kruskall-Wallis Test (Chi2 = 230.84, p-value <0.001, df = 2; followed by Dunn's posthoc test with Benjamini-Hochberg p-value correction).

Table S1: Statistical analyses showing the absence of significant differences between animals within each condition.

Number figure	Statistical tests	p-value
Fig. 1E	Fisher's Exact	p-value = 0.6958
Fig. 1F	Fisher's Exact<br>Fisher's Exact	Migration: p-value = 1<br>Pause: p-value = 0.8061
Fig. 1G	Fisher's Exact<br>Fisher's Exact	Internalized cilium: p-value = 0.7811<br>Emerged cilium: p-value = 1
Fig. 2D	Fisher's Exact	p-value = 0.6482
Fig. 2E	Fisher's Exact<br>Fisher's Exact	Migration: p-value = 0.7431<br>Pause: p-value = 0.8581
Fig. 2F	Fisher's Exact<br>Fisher's Exact	Internalized cilium: p-value = 0.3853<br>Emerged cilium: p-value = 0.2184
Fig. 2G	Pearson's Chi-squared test<br>Pearson's Chi-squared test<br>Pearson's Chi-squared test<br>Fisher's Exact	WT_NoFlow: p-value = 0.3613<br>WT_Flow: p-value = 0.7854<br>LvmiRNeg_Flow: p-value = 0.6062<br>LvmiRGPR161_Flow: p-value = 0.1474

Movie S1.
Three-dimensional reconstruction image of a neuroblast in the RMS having an internalized primary cilium. Blue, white, and magenta represent cell contour, Arl13b (ciliary marker), and GPR161, respectively. Scale bar: 3µm.

Movie S2.
Three-dimensional reconstruction image of a neuroblast in the RMS having an externalized primary cilium. Blue, white, and magenta represent cell contour, Arl13b (ciliary marker), and GPR161, respectively. Scale bar: 3µm.

Movie S3.
Time-lapse imaging of miRNeg-GFP electroporated neuroblasts in rostral migratory stream (RMS). The arrow represents the direction of migration, from the V/SVZ to the olfactory bulb. Scale bar: 50 µm.

Movie S4.
Time-lapse imaging of miRGPR161CDS-GFP electroporated neuroblasts in rostral migratory stream (RMS). The arrow represents the direction of migration, from the V/SVZ to the olfactory bulb. Scale bar: 50 µm.

Movie S5.
Time-lapse imaging of miRGPR161UTR-GFP + GPR161wt electroporated neuroblasts in rostral migratory stream (RMS). The arrow represents the direction of migration, from the V/SVZ to the olfactory bulb. Scale bar: 50 µm.

Movie S6.
Time-lapse imaging of miRGPR161UTR-GFP + GPR161H8del electroporated neuroblasts in rostral migratory stream (RMS). The arrow represents the direction of migration, from the V/SVZ to the olfactory bulb. Scale bar: 50 µm.

Movie S7.
Three-dimensional reconstruction image of a neuroblast from V/SVZ in 2D culture having an internalized primary cilium. Blue, white, and magenta represent cell contour, Arl13b (ciliary marker), and GPR161, respectively. Scale bar: 3µm.

Movie S8.
Three-dimensional reconstruction image of a neuroblast from V/SVZ in 2D culture having an externalized primary cilium. Blue, white, and magenta represent cell contour, Arl13b (ciliary marker), and GPR161, respectively. Scale bar: 3µm.

Movie S9.
Time-lapse imaging of 2D-cultured neuroblasts from the V/SVZ under no-flow condition. Tracks highlight neuroblasts with a distinct neuroblast migrating morphology, selected for migration analysis. Scale bar: 50 µm.

Movie S10.
Time-lapse imaging of 2D-cultured neuroblasts from the V/SVZ under flow condition (0.13Pa). Tracks highlight neuroblasts with a distinct neuroblast migrating morphology, selected for migration analysis. Flow is directed from the top to the bottom of the field of view. Scale bar: 50 μm.

Movie S11.
Time-lapse imaging of 2D-cultured neuroblasts from the V/SVZ infected with LvMiRNeg and under flow condition (0.13Pa). Tracks highlight neuroblasts with a distinct neuroblast migrating morphology, selected for migration analysis. Flow is directed from the top to the bottom of the field of view. Scale bar: 50 μm.

Movie S12.
Time-lapse imaging of 2D-cultured neuroblasts from the V/SVZ infected with LvMiRGPR161 and under flow condition (0.13Pa). Tracks highlight neuroblasts with a distinct neuroblast migrating morphology, selected for migration analysis. Flow is directed from the top to the bottom of the field of view. Scale bar: 50 μm.

Movie S13.
Time-lapse imaging of a control neuroblast in in rostral migratory stream (RMS) electroporated with EpacSh187 cAMP biosensor. cAMP imaging analyzed with the plugin FRETratioFx on ImageJ to create a ratio image of non-FRET over FRET fluorescence intensity, which reports biosensor cAMP activation level. The ratio for each pixel is calculated and converted into a hue value. Scale bar: 5 μm.

Movie S14.
Time-lapse imaging of a control neuroblast in rostral migratory stream (RMS) electroporated with EpacSh187 cAMP biosensor + miRGPR161CDS-Tdto. cAMP imaging was analyzed with the plugin FRETratioFx on ImageJ to create a ratio image of non-FRET over FRET fluorescence intensity, which reports biosensor cAMP activation level. The ratio for each pixel is calculated and converted into a hue value. Scale bar: 5 μm.

Movie S15.
Time-lapse imaging of Nde1PMutant + miRNeg-GFP electroporated neuroblasts in rostral migratory stream (RMS). The arrow represents the direction of migration, from the V/SVZ to the olfactory bulb. Scale bar: 50 μm.

Movie S16.
Time-lapse imaging of Nde1pmimic + miRGPR161CDS-GFP electroporated neuroblasts in rostral migratory stream (RMS). The arrow represents the direction of migration, from the V/SVZ to the olfactory bulb. Scale bar: 50 μm.
